# Design of Friction, Morphology, Wetting, and Protein Affinity by Cellulose Blend Thin Film Composition

**DOI:** 10.3389/fchem.2019.00239

**Published:** 2019-05-03

**Authors:** Caterina Czibula, Gundula Teichert, Maximilian Nau, Mathias Hobisch, Chonnipa Palasingh, Markus Biesalski, Stefan Spirk, Christian Teichert, Tiina Nypelö

**Affiliations:** ^1^Institute of Physics, Montanuniversität Leoben, Leoben, Austria; ^2^Christian Doppler Laboratory for Fiber Swelling and Paper Performance, Graz University of Technology, Graz, Austria; ^3^Institute of Paper, Pulp and Fiber Technology, Graz University of Technology, Graz, Austria; ^4^Macromolecular Chemistry and Paper Chemistry, Technical University Darmstadt, Darmstadt, Germany; ^5^Division of Applied Chemistry, Department of Chemistry and Chemical Engineering, Chalmers University of Technology, Gothenburg, Sweden; ^6^Wallenberg Wood Science Center, Gothenburg, Sweden

**Keywords:** blend films, spinodal decomposition, cellulose, friction, protein adsorption, adhesion

## Abstract

Cellulose derivate phase separation in thin films was applied to generate patterned films with distinct surface morphology. Patterned polymer thin films are utilized in electronics, optics, and biotechnology but films based on bio-polymers are scarce. Film formation, roughness, wetting, and patterning are often investigated when it comes to characterization of the films. Frictional properties, on the other hand, have not been studied extensively. We extend the fundamental understanding of spin coated complex cellulose blend films via revealing their surface friction using Friction Force Microscopy (FFM). Two cellulose derivatives were transformed into two-phase blend films with one phase comprising trimethyl silyl cellulose (TMSC) regenerated to cellulose with hydroxyl groups exposed to the film surface. Adjusting the volume fraction of the spin coating solution resulted in variation of the surface fraction with the other, hydroxypropylcellulose stearate (HPCE) phase. The film morphology confirmed lateral and vertical separation and was translated into effective surface fraction. Phase separation as well as regeneration contributed to the surface morphology resulting in roughness variation of the blend films from 1.1 to 19.8 nm depending on the film composition. Friction analysis was successfully established, and then revealed that the friction coefficient of the films could be tuned and the blend films exhibited lowered friction force coefficient compared to the single-component films. Protein affinity of the films was investigated with bovine serum albumin (BSA) and depended mainly on the surface free energy (SFE) while no direct correlation with roughness or friction was found. BSA adsorption on film formed with 1:1 spinning solution volume ratio was an outlier and exhibited unexpected minimum in adsorption.

## Introduction

Spinodal decomposition of polymer blends can generate thin films with multi-phase surface composition, and often, complex morphology (Heriot and Jones, 2005). Lateral separation of the phases on a substrate results in patterns where islands of one phase reside within the other phase. Vertical separation can lead to heterogeneous distribution in the film's z-direction induced by surface energy differences of the components and can influence the surface morphology (Karim et al., 1998).

An intriguing area of research is the exploration of blend thin films obtained with spin coating. During the spin coating step, macroscopically homogeneous solutions of two polymers phase separate into the domains (Dalnoki-Veress et al., 1997). The ratio of the two polymers, molecular weight, solvent and spin coating conditions define the feature sizes and shapes (Xue et al., 2012). Synthetic polymer phase separation is exploited in coatings and organic electronics (Halls et al., 1995), while for biopolymer blend thin films their utilization and fundamentals, are still in their origins.

Cellulose synthetized by nature is notorious for insolubility having only few one-component solvents such as N-methylmorpholine-N-oxide (Medronho et al., 2012). In industrial fiber production, cellulose insolubility has been circumvented by derivatization followed by conversion to cellulose commonly referred to as regeneration. Similar approaches have been applied in thin film manufacturing involving derivatization, dissolution, film formation, and regeneration of the derivatized film to cellulose (Schaub et al., 1993; Kontturi et al., 2003a,b). Spin coating, and propagation of repeating submicron patterns, requires volatile and good solvents for the blend components. Trimethylsilyl cellulose (TMSC) dissolved in toluene or chloroform has been used for single-component cellulose films as well as for blend films (Kontturi et al., 2009, 2010; Nyfors et al., 2009; Niegelhell et al., 2016, 2017; Strasser et al., 2016). Regeneration takes place upon exposure to hydrochloric acid vapor (Schaub et al., 1993).

Cellulose in products is often an inert component intended to protect or seal, such as package products, or to carry functionalities as in paper-based diagnostics (Pelton, 2009). As a substrate it can be used to accommodate follow-up chemistries, for example, to bind peptides to further tune it for specific protein or antibody affinity and sensing of biological molecules (Orelma et al., 2012a,b; Zhang et al., 2013). In addition to detection, protein adsorption is a way to design medical materials or to control fouling of surfaces. The inhibition of protein deposition, on the other hand, is an asset when bacterial growth and biofilm formation are required to be blocked.

Biofilm formation on surfaces takes place in two phases where the first one includes reversible physical attachment of bacteria and the second irreversible, cellular phase. Surface roughness, wetting and surface configuration have been identified as key parameters for bacterial adhesion (An and Friedman, 1998). The effect of hydrophobicity is directly in connection with the properties of the bacteria while with respect to roughness there is direct evidence that increase in roughness—and hence, in the surface area—promotes bacterial adhesion. Roughness is different from surface configuration that refers to patterning on the surface. The periodicity and size of surface patterns has been found to be a parameter to inhibit bacterial adhesion. While bacteria preferentially adhere to irregularities that conform to their size since this maximizes bacteria-surface area (Katsikogianni and Missirlis, 2004), there is evidence that specific surface pattern design can prevent the attachment. A pattern mimicking a skin of shark was able to significantly reduce biofilm formation (Chung et al., 2007). The key parameters of the biofilm inhibiting films are non-random patterns with a hierarchy where the size is optimized to the size of a specific bacteria (Schumacher et al., 2007a,b). Here, we apply spinodal separation to generate periodical cellulose blend film hierarchies and evaluate the morphology and configuration and discuss their relation to antifouling surfaces.

Atomic force microscopy (AFM) is a non-destructive analytical tool applying low forces and is therefore suited for nanoscale characterization of soft polymer and biological surfaces. Apart from morphology studies, phase contrast in tapping mode imaging (Tamayo and Garcia, 1996), chemical contrast with functionalized AFM tips (Frisbie et al., 1994), and mechanical contrasts (Chyasnavichyus et al., 2015; Kocun et al., 2017) are established investigation routines. Phase and adhesion contrast measurements have been explored also on cellulosic films to achieve chemical contrast with functionalized probes (Ganser et al., 2016). Friction behavior of blend films can be studied on the nanoscale by friction force microscopy (FFM) (Mate et al., 1987; Marti et al., 1990; Meyer and Amer, 1990). In FFM, the AFM tip scans in contact mode normal to the cantilever's long axis, and the resulting cantilever torsion is related to the friction coefficient. Friction contrast for polymers by FFM has been demonstrated for phase-separated thin organic films (Overney et al., 1992, 1994) where differences in the friction signal between hydrocarbon and fluorocarbon containing domains were found. More recently, FFM was employed to correlate friction to viscoelastic relaxation (Hammerschmidt et al., 1996; Sondhauß et al., 2015) and to characterize photoreactive organic surface patterns of spin casted thin films (Hlawacek et al., 2009; Shen et al., 2014). Polyisoprene and polystyrene blend coatings were recently reported to show an unexpected tribological synergy (Emerson et al., 2017). By varying the composition of the blend films, it was possible to tune the tribological properties and achieve friction coefficients which are much lower than for the pure films.

A blend of TMSC (regenerable to cellulose) and hydroxypropylcellulose stearate (HPCE) resulted in micropatterned films with varying aliphatic surface concentration. HPCE, a cellulose derivative, exhibits long alkyl side chains which may act as brushes and in combination with cellulose impact, wetting, adhesion, and protein adsorption. The surface morphology, roughness and lateral correlation length, of the films was quantitatively studied using AFM. The friction behavior was analyzed by FFM and compared to the adhesive properties of the surfaces of the pure and blend films obtained from AFM force spectroscopy. Finally, the comprehensive information obtained by surface characterization was employed to determine the influence of adhesive and tribological surface properties of the cellulose-HPCE blend films on bovine serum albumin (BSA) adsorption investigated by surface plasmon resonance spectroscopy (SPR) and Quartz Crystal Microbalance with Dissipation monitoring (QCM-D).

## Materials and Methods

### Materials

**Figure 1:**
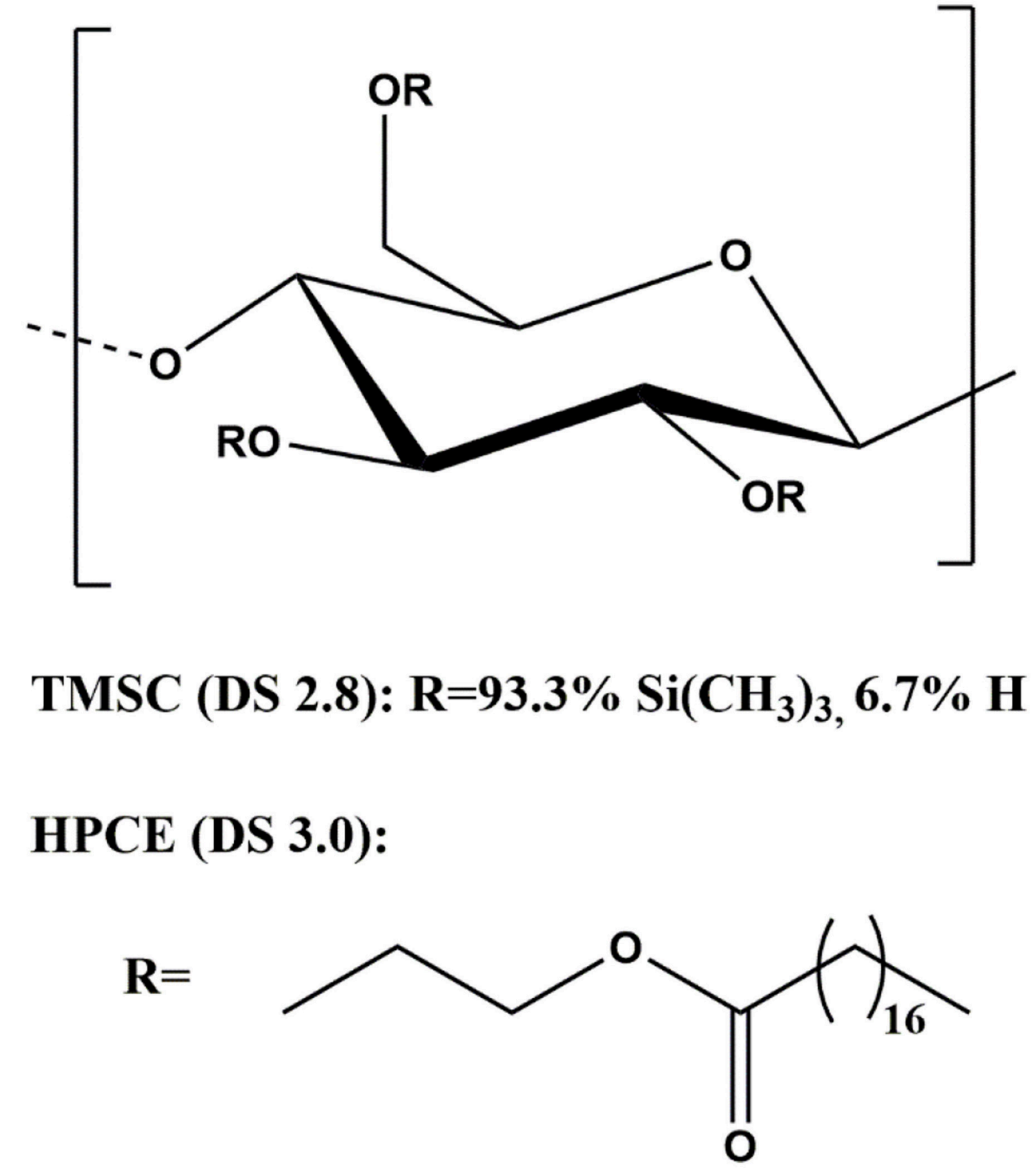
Structure of trimethylsilyl cellulose and hydroxypropylcellulose stearate.

Hydroxypropylcellulose stearate (HPCE, M_w_ 134,700 g mol^−1^, M_n_ 107,400 g mol^−1^, polydispersity index 1.25, degree of substitution (DS) 3.0) was synthesized according to a literature protocol (Nau et al., 2018). Trimethylsilyl cellulose (TMSC, From Avicel, M_w_ 185,000 g mol^−1^, M_n_ 30,400 g mol^−1^, polydispersity index 6.1, DS 2.8) was purchased from TITK (Thuringian Institute of Textile and Plastics Research, Germany). The structures are shown in Figure 1. Chloroform (99.3%), disodium phosphate heptahydrate (Na_2_HPO_4_·7H_2_O), sodium dihydrogen phosphate monohydrate (NaH_2_PO_4_·H_2_O), hydrochloric acid (37%), and bovine serum albumin (BSA) were purchased from Sigma-Aldrich and were used as received. Silicon wafers used as film substrates were cut 1 cm × 2 cm. Surface plasmon resonance (SPR) gold sensor slides (CEN102 AU) were purchased from Cenibra (Germany). Milli-Q water (resistivity = 18.2 Ω^−1^ cm^−1^ at 25°C) from a Millipore water purification system (Millipore, U.S.A.) was used for contact angle, SPR, and QCM-D investigations.

### Substrate Cleaning and Film Preparation

The film substrates were cleaned by immersing them in an *in-situ* produced peroxymonosulfuric acid containing H_2_O_2_ (30 wt%)/ H_2_SO_4_ (1:3 v/v) for 10 min for SPR spectroscopy slides or 30 min for silicon wafers, respectively. After rinsing with deionized water, the wafers were dried with nitrogen gas, rinsed and stored in deionized water. TMSC and HPCE were dissolved in chloroform in a concentration of 0.75 wt%, using a water bath heated to 30°C, and 120 h on a magnetic stirrer. Right before use the solutions were filtered through 0.45 μm PVDF filters (Chromafil) and mixed in volumetric ratios labeled further on as TMSC:HPCE 1:0, 1:3, 1:1; 3:1, and 0:1. A volume of 100 μl was used for spin coating and operated for 60 s with an acceleration of 2,500 rpm s^−1^ and a speed of 4,000 rpm.

The conversion of TMSC into cellulose was implemented in a polystyrene petri dish (5 cm in diameter) containing 3 ml of 10 wt% HCl. The substrates were exposed to HCl vapor for 12 min. The regeneration of cellulose from TMSC was verified by contact angle and ATR-IR measurements (Alpha FT-IR spectrometer, Bruker, U.S.A.) using an attenuated total reflection attachment and obtaining spectra between 4,000 and 400 cm^−1^ with 48 scans and a resolution of 4 cm^−1^. The data was analyzed with OPUS 4.0 software.

### Profilometry

Thickness of the thin films was determined by scratching the films with a scalpel and measuring the profile of a scan length of 1,000 μm and a duration of 3 s using a DETAK 150 Stylus Profiler from Veeco (Bruker, USA) on a hydraulic balanced stone table with a diamond stylus with a radius of 12.5 μm and a force of 3 mg. Three films of each sample were measured at 6 locations before and after regeneration. Film thickness and film roughness was calculated from the resulting profile using Software Vision 64.

### Contact Angle (CA) and Surface Free Energy (SFE) Determination

Static contact angle measurements were performed with a Drop Shape Analysis System DSA100 (Krüss GmbH, Germany) with a T1E CCD video camera (25 frames per second) and the DSA1 v 1.90 software. All measurements were performed at least three times on minimum two manufactured films with Milli-Q water and diiodomethane using a droplet size of 3 μL and a dispense rate of 400 μL min^−1^. Static CAs were calculated with the Young-Laplace equation, and the SFE was determined with the Owen-Wendt-Rabel-Kaelble (OWRK) method. Surface tension of 50.80 and 72.80 mN m^−1^ for diidomethane and water were used, respectively.

### Adsorption Experiments

A phosphate buffer containing 8.1 mM disodium phosphate, 1.9 mM sodium phosphate and 100 mM sodium chloride at pH 7.4 was used to carry out the adsorption experiments of BSA on the films. Surface Plasmon Resonance Spectroscopy (SPR) was performed with a MP-SPR Navi 200 from Bionavis Ltd (Finland), using 785 nm laser in both measurement channels. The attached autosampler MP-SPR Navi 210A was set to 20 μl min^−1^ flow rate. The equilibration of the thin films was observed by measuring the spectra with full angular scan (39–78°) and scan speed of 8° s^−1^ at 24.5° and plot the SPR-angle over time. A concentration of 1.0 mg ml^−1^ of BSA was dissolved in the buffer and exposed to the thin films for 10 min. Adsorbed mass (Γ) was calculated with the de Feijter equation,

(1) $$\begin{array}{r} \Gamma =\frac{\Delta \Theta^{*}\kappa^{*}d_{p}}{dn/dc} \end{array}$$

using the refractive index increment (dn/dc) 0.182 cm3 g^−1^. The ΔΘ is the angular response of the surface plasmon resonance. For thin layers (<100 nm), k × d_p_ can be considered constant and can be obtained by calibration of the instrument by determination of the decay wavelength l_d_. Here it was 1.09 × 10^−7^ cm/° (at 670 nm) and 1.9 × 10^−7^ cm/° (at 785 nm) in aqueous systems.

Quartz Crystal Microbalance and Dissipation (QCM-D) instrument (model E4) from Q-Sense (Sweden) was used with gold sensors purchased from QuartzPro (Sweden). The attached peristaltic pump was set to 0.1 ml min^−1^. Adsorption was performed in the same conditions as the SPR analyses. The data was analyzed using Johannsmann modeling (Johannsmann et al., 1992; Naderi and Claesson, 2006).

### Atomic Force Microscopy

Most AFM and all FFM measurements were acquired using an Asylum Research MFP-3D AFM (USA). The instrument is equipped with a closed-loop planar x-y-scanner with a scanning range of 85 μm × 85 μm and a z-range of 15 μm. The tapping mode AFM images were recorded with standard silicon probes (Olympus AC160TS, cantilever spring constant ~30 N m^−1^, tip radius ~10 nm). The measurements were obtained in ambient conditions at 50 ± 8% relative humidity and a temperature of 22 ± 1°C. Topography images of three independent positions were recorded for each sample. All the data was processed in the open-source software Gwyddion (Necas and Klapetek, 2012). For the 5 μm × 5 μm images, a roughness analysis (Teichert, 2002) was performed by calculating the 1D height-height correlation function:

(2) $$\begin{array}{r} C\left(x\right)=\;\langle \left[z\left(x_{0}+x\right)-\;\langle z\rangle \right]\left[z\left(x_{0}-\;\langle z\rangle \right)\right]\rangle \end{array}$$

of each scan line and then averaging over all lines. The resulting values were fitted with the function:

(3) $$\begin{array}{r} C\left(x\right)=\;\sigma^{2}\;e^{{-\left(\frac{\left|x\right|}{\xi }\right)}^{2\alpha }} \end{array}$$

The parameters σ, ξ, and α are used to characterize the surface roughness (Teichert, 2002). The σ denotes the root mean square (RMS) roughness, i.e., the standard deviation of the height values, which is a common measure for the vertical roughness. The lateral correlation length ξ describes the lateral fluctuation of the height values and α is the so-called Hurst parameter or roughness exponent. It determines the shape of C(x) and quantifies the jaggedness of the surface.

For FFM, which is recorded in contact mode, NT-MDT CSG10/Au probes with a tip radius of about 30 nm and a low cantilever spring constant of 0.1 N m^−1^ were employed. Images with frame size of 5 μm × 5 μm were obtained with a constant scan speed of 2.5 μm s^−1^. A vertical force of about 10 nN was applied during the measurements. For acquisition of an FFM image, standard contact mode AFM scan including the lateral trace and retrace channel were recorded. The raw lateral signals were converted to friction images by subtracting the lateral retrace from the lateral trace signal and dividing it by two for each image (Kalihari et al., 2010; Shen et al., 2014). This eliminates topography artifacts and a possible offset.

The quantitative interpretation in terms of friction coefficient is not straightforward, and literature includes several calibration methods (Klapetek, 2018). Lateral force sensitivity calibration was done here according to the wedge calibration method of Varenberg et al. (2003). Quantitative friction images were obtained by multiplying the resulting friction image data with the lateral force sensitivity using the Gwyddion software.

AFM force spectroscopy measurements to investigate the adhesion properties of the film surfaces were performed with a scan rate of 2 Hz and a force distance of 0.5 μm. For these measurements, HSC60 probes from Team Nanotec (Germany) were used which have a cantilever spring constant of about 50 N/m and a tip radius of 60 nm. Here, 32 × 32 px^2^ maps were obtained on 5 μm × 5 μm topography scans.

A Veeco Multimode Quadrax MM AFM (Bruker, USA) in tapping mode using standard silicon probes (NCH-VS1-W, NanoWorld AG) was used for recording film topography after they were rinsed with chloroform.

### Surface Morphology and Area Determination

To quantify the observed surface features for the individual blend films, ten individual cross-sections of the features were obtained from the topography images for each film with the Gwyddion software to determine the height and width of the features. The values are given as mean ± standard deviation.

The AFM topography images were used for calculating effective surface fractions of the blend films. These are referred to as “effective surface fraction” or “surface fraction” later in the manuscript. The film component fraction derived from spinning solution is referred to as “volume fraction” or “volume ratio.” Masked surfaces were evaluated by the surface area estimate method in Gwyddion, computed by simple triangulation that considers heights and spatial relations in the surface. For this purpose, additional points were added in-between four neighboring points using the mean values of these pixels. Four triangles are formed, and the surface area is approximated by summing their areas. Masking was done by threshold in *z*-value and adjusting with a pen tool. Single noise pixel was removed by grain filtering function. For films that showed a reliable phase contrast in the tapping mode topography analysis, the masks were determined from the phase information.

## Results and Discussion

**Figure 2:**
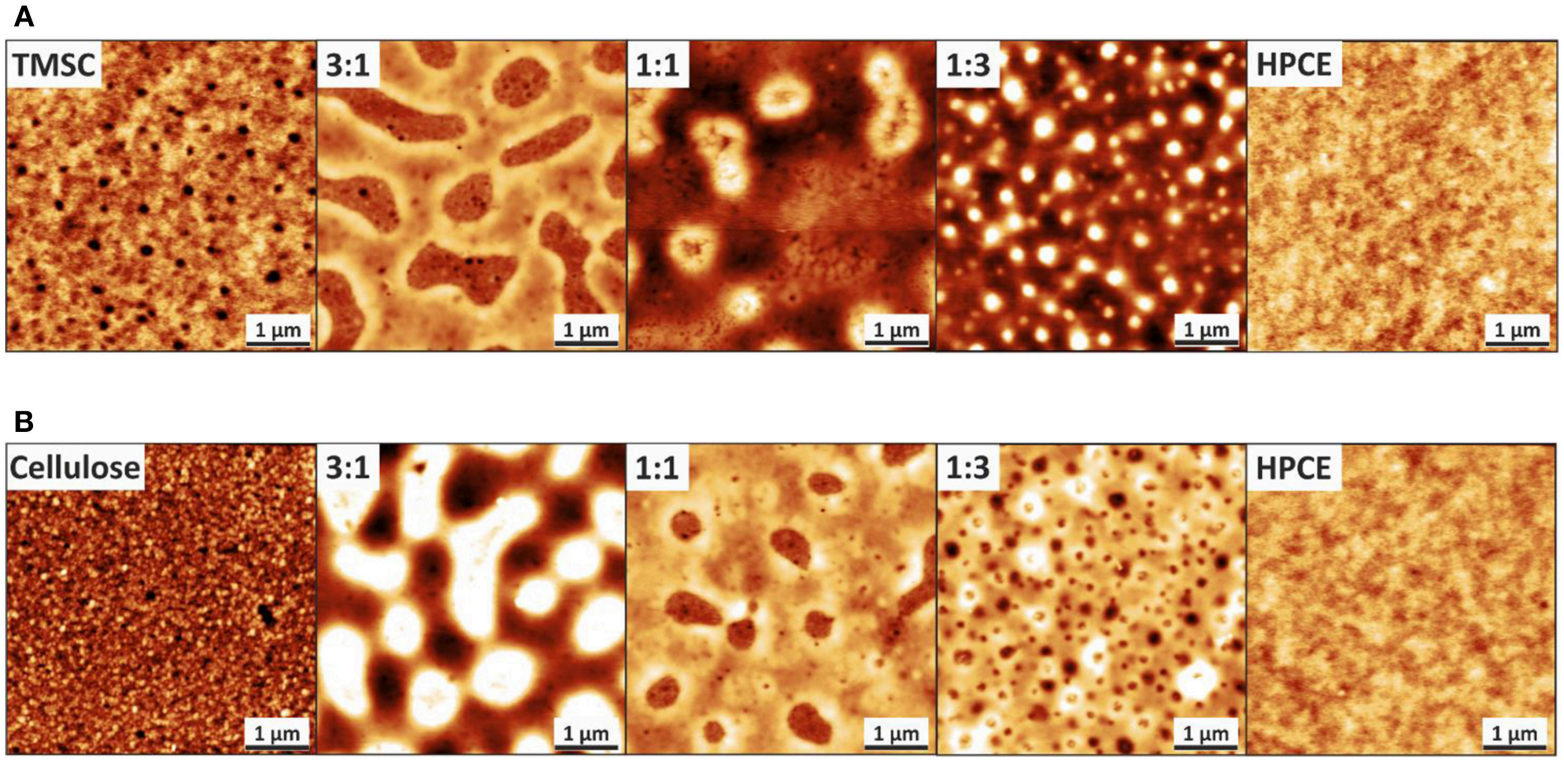
AFM topography images of **(A)** TMSC:HPCE and **(B)** cellulose:HPCE blend films. The z-scale for the neat films is 10 nm and for all the blend films 60 nm. The z-directional height increases with increasing brightness.

Spin coated TMSC and HPCE films formed into smooth one-component film morphologies—indicating a uniform film formation—while the blend films containing both derivatives resulted in films with spinodal decomposition and are presented via three composition ratios, 3:1, 1:1, and 1:3 (Figure 2A). These TMSC:HPCE blend film surfaces consisted of domains which either formed cavities (TMSC:HPCE 3:1) or protrusions in lower sub-micrometer range. Protrusions with height 35 ± 10 nm and width of 780 ± 130 nm were determined for TMSC:HPCE 1:1 and of height of 55 ± 10 nm and width of 340 ± 50 nm for TMSC:HPCE 1:3. The protrusions increased in height with increasing HPCE content but decreased in lateral size.

The regeneration of TMSC into cellulose is accompanied by the cleavage of the silyl groups and simultaneous formation of hydrogen bonds of the created hydroxyl groups. This led to densification and reduction in film thickness (Figure S1), similar to reported elsewhere (Wolfberger et al., 2015), of the regenerated parts and consequently, more complex morphology (Figure 2B). HPCE is unaffected by the treatment (compare Figures 2A,B). After conversion to cellulose, the blend film cellulose fraction mass was reduced which translated into an inverted morphology compared to the TMSC:HPCE blend films. The cellulose:HPCE 3:1 surface was characterized by protrusions with arbitrary size and a height of 40 ± 10 nm, whereas the cellulose:HPCE 1:1 and 1:3 films contained cavities which decrease in lateral size. The 1:1 sample showed random-shaped surface features with a lateral width of 660 ± 80 nm, but the more spherical-shaped features on the 1:3 blend film surface had a width of only 250 ± 25 nm. An overview of this data is presented in Table 1.

**Table 1:** RMS roughness and lateral correlation length of the roughness of the blend films prior (TMSC:HPCE) and after (cellulose:HPCE) regeneration.

	**RMS roughness σ [nm]**	**Lateral correlation length ξ [nm]**		**RMS roughness σ [nm]**	**Lateral correlation length ξ [nm]**
TMSC	1.50 ± 0.05	95 ± 5	Cellulose	1.50 ± 0.20	55 ± 10
3:1	7.60 ± 0.30	185 ± 15	3:1	19.80 ± 0.30	310 ± 10
1:1	12.60 ± 0.80	320 ± 20	1:1	8.00 ± 0.40	180 ± 5
1:3	14.15 ± 0.75	145 ± 10	1:3	10.20 ± 0.10	155 ± 5
HPCE	1.10 ± 0.05	120 ± 10	HPCE	1.10 ± 0.10	140 ± 10

*The data is obtained from analysis of the 5 μm × 5 μm topography images*.

**Figure 3:**
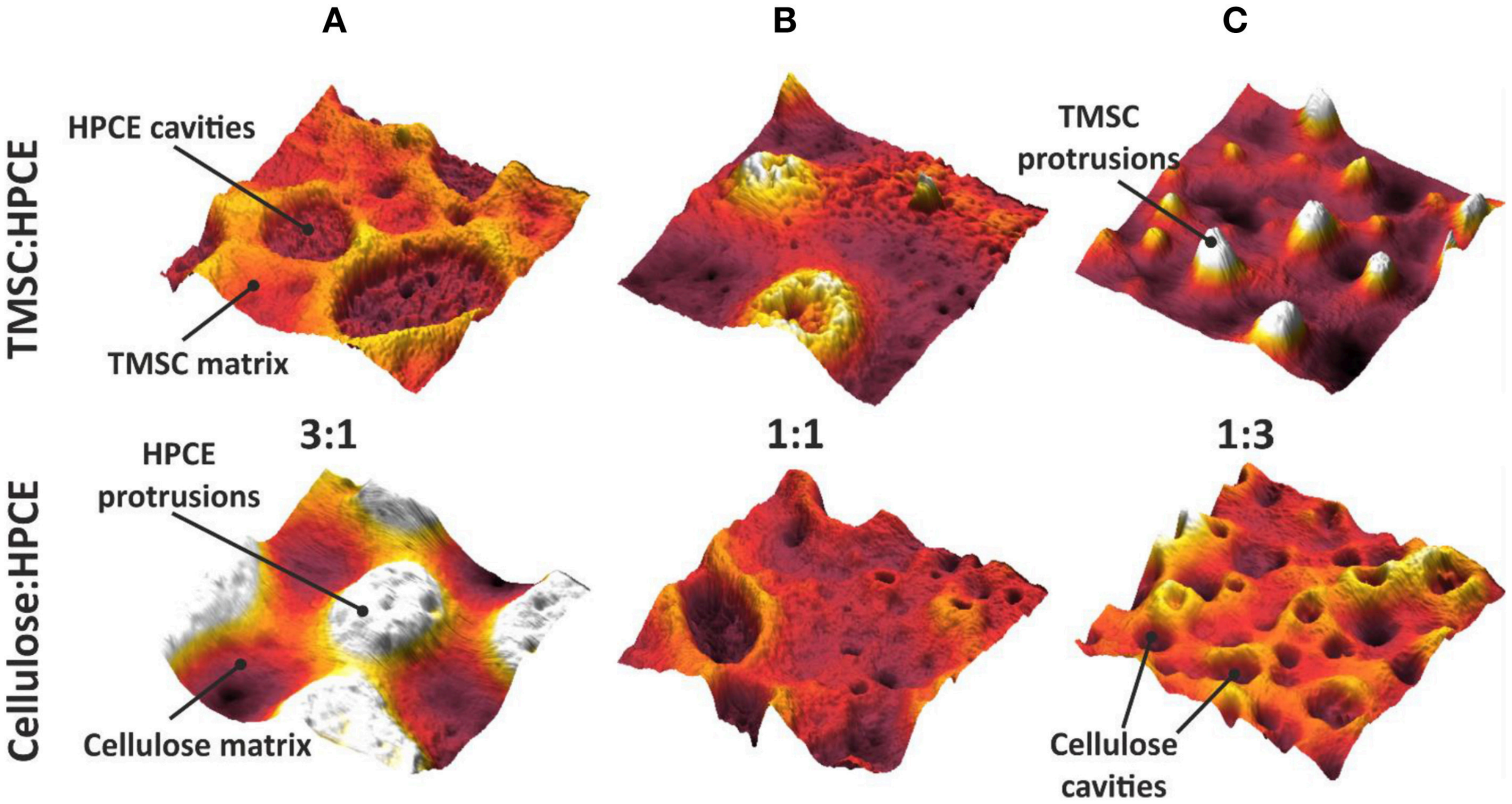
Three-dimensional (2 μm × 2 μm) AFM topography images of the TMSC:HPCE (top row) and cellulose:HPCE (bottom row) blend films. **(A)** 3:1, **(B)** 1:1, and **(C)** 1:3. The z-scale is 60 nm in all cases.

The phase assignment was confirmed by treating the cellulose:HPCE blend films with chloroform that removed the HPCE phase. The remaining cellulose matrix was visualized and confirmed that the cellulose was the continuous phase in 3:1 blends, and the discontinuous one in 1:1 and 1:3 films (see Figure S2A). The phase assignments were incorporated in three-dimensional AFM topography images (Figure 3). The TMSC:HPCE 3:1 blend film consisted of a continuous TMSC matrix with HPCE cavities. Regeneration of the TMSC to cellulose resulted in a shrinkage of the TMSC domains. Therefore, the HPCE domains were protruding from the surface of the cellulose:HPCE blend films. With increasing HPCE amount, the matrix increasingly consisted of HPCE and the TMSC domains formed protrusions, which collapsed during cellulose regeneration (Figures 3B,C).

The visualization of the blend film after removal of the HPCE phase of 1:1 film did not only reveal the cellulose left behind in islands (Figure S2A). Lateral phase separation is chiefly responsible for the phase separation patterns that were observed upon the spinodal decomposition (Figure 2). This can take place independently or simultaneously with vertical separation which results in heterogeneous layer formation in the z-direction (Karim et al., 1998; Heriot and Jones, 2005). Dissolution of the HPCE phase revealed the cellulose skeleton left behind creating roughness beyond the apparent cellulose islands (Figure S2A).

**Figure 4:**
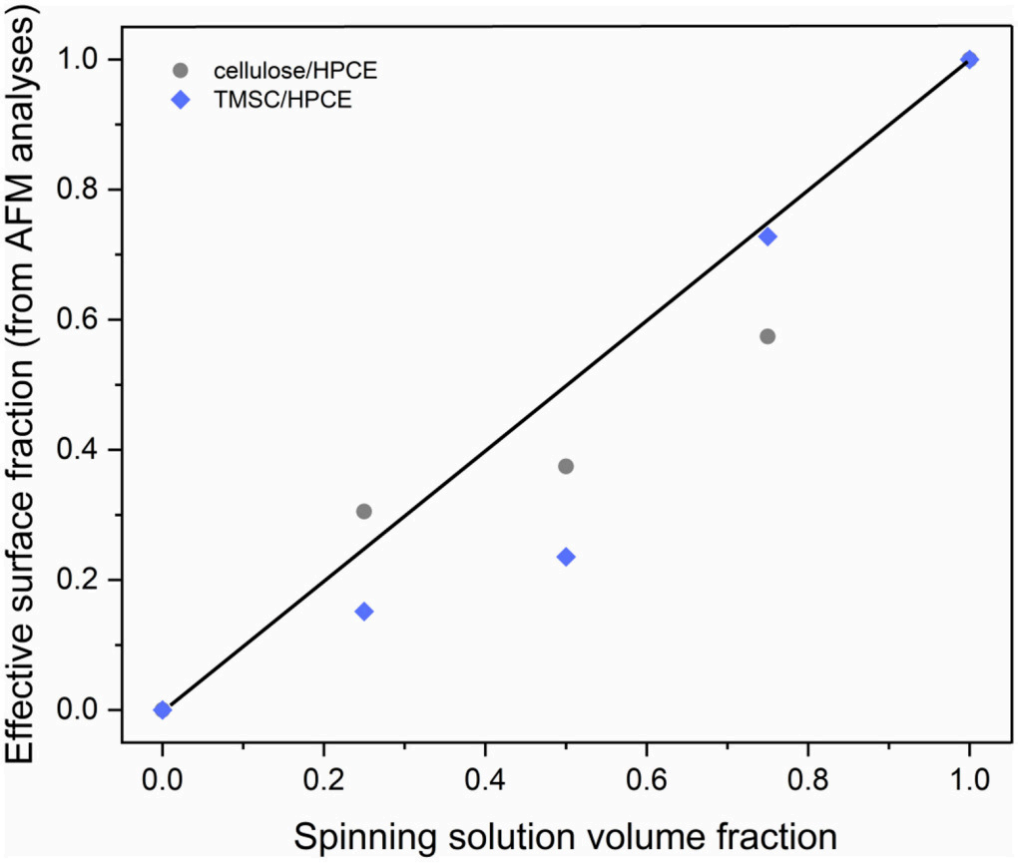
Relationship of the TMSC:HPCE blend spinning solution volume fraction and experimentally determined effective surface fraction of the TMSC:HPCE (diamonds) and cellulose:HPCE films (circles). The black line represents identical volume and surface fraction.

The vertical separation and z-directional morphology evidently had an impact on the surface composition of the films meaning that the volume fraction did not necessarily equal to a surface area fraction. We used the AFM analyses to calculate effective surface area taking into consideration the surface roughness. The calculated surface areas were converted into surface fraction so that the TMSC or regenerated cellulose phase area was divided by the total area. The surface area could not be solely determined from the topography analysis (Figure S2B) but required phase imaging to reveal additional cellulose domains (Figure S2C). These were counted in to the cellulose surface fraction. Neither the TMSC:HPCE (diamonds) nor cellulose:HPCE (circles) surface fraction correlated directly with the volume fraction of the polymer blends used in spin coating (Figure 4). The surface fraction decreased upon regeneration in the case of high cellulose dominant blend films and increased with HPCE dominant ones. The differences in surface fraction of cellulose between the 1:3 and 1:1 sample is small (30% vs. 37%), but the resulting microstructures are very different (Figures 3B,C, lower row).

The roughness σ of the resulting blend thin films increased by factors of 4 to 20 compared to the neat films (Table 1). For the TMSC:HPCE blend films, the 1:3 composition featured the largest σ while after regeneration by HCl vapors, the cellulose:HPCE 3:1 film exhibited largest σ. The lateral correlation lengths ξ were lowest for the pure films. For the blend films, ξ increased by up to a factor of 3. TMSC:HPCE 1:1 and cellulose:HPCE 3:1 showed the highest values.

Derivatization of cellulose with the hydroxypropyl stearyl side chain (Figure 1) modifies the hydrophobicity of the molecule and this should reflect to the water contact angle and the surface free energy (SFE) of the films. The water contact angle of the cellulose film was 36.6 ± 0.3 degrees, TMSC film 94.6 ± 0.1 degrees, and that of the HPCE film 77.9 ± 0.5 degrees. The SFE of the blend films increased with increasing cellulose fraction (Figure S3) while prior to regeneration SFE was below 30 mJ m^−2^ for all the films. Consequently, the polar contribution decreased with the same trend (Figure S4).

### Correlation of Friction Coefficient, Adhesion Force, Surface Roughness to Surface Free Energy and Protein Affinity of the Cellulose/HPCE Blend Films

**Figure 5:**
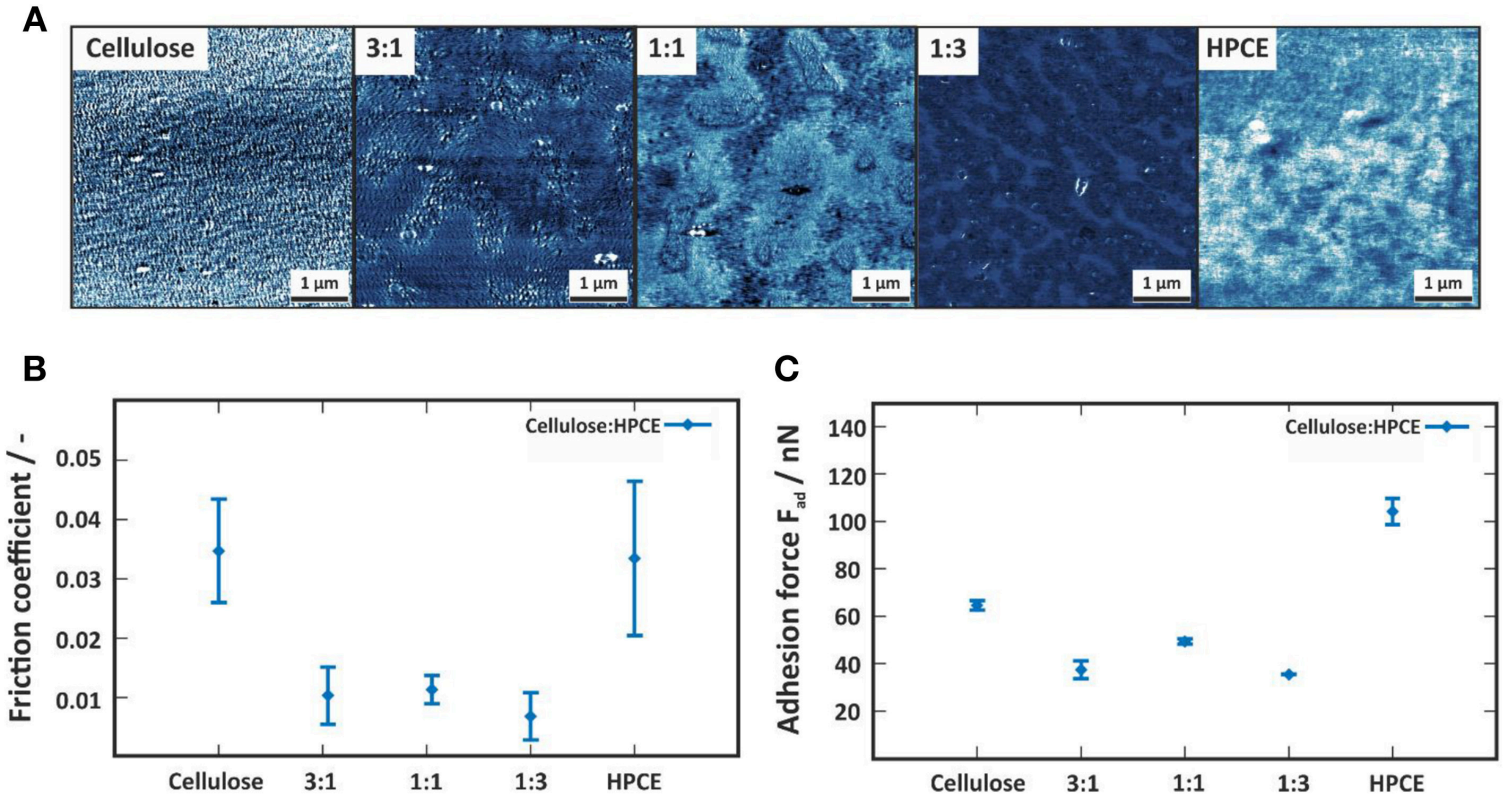
**(A)** Contact mode friction force images of the cellulose, cellulose:HPCE and HPCE films. The z-scale of all images is 5 mV. **(B)** Friction coefficient and **(C)** adhesion force for the cellulose, cellulose:HPCE and HPCE films.

The contact mode FFM images (Figure 5A) enclose the distinct differences between the neat and the cellulose/HPCE blend films' friction (indicated by the contrast differences). The average friction coefficients (Klunsner et al., 2010) were lower for the blend films than for the pure films (Figure 5B). The same applied for the adhesion forces (Figure 5C) where the blends featured lower values (20–50 nN) than the cellulose and HPCE films (65 ± 2 nN, and 104 ± 5 nN, respectively). It should be noted here that friction force data is in good agreement with those reported for cellulose spheres interacting with modified silica surfaces in a similar applied force range (~5 nN) (Bogdanovic et al., 2001).

### Non-specific Protein Deposition—BSA Adsorption

**Figure 6:**
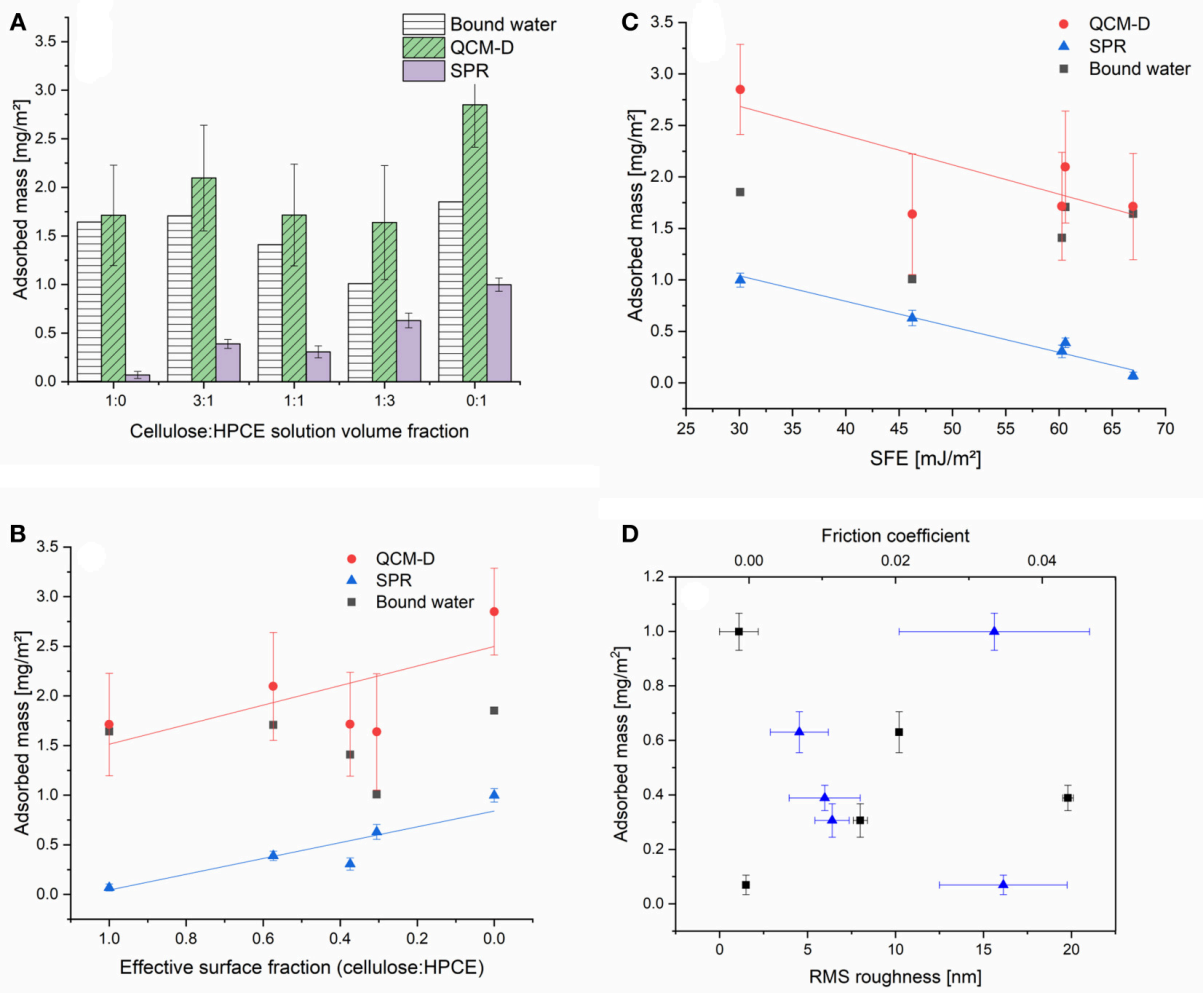
**(A)** Adsorbed mass (mg m^−2^) of BSA on the cellulose:HPCE blend films determined by SPR and QCM-D. Adsorbed masses with respect to the **(B)** surface fraction, and **(C)** surface free energy. **(D)** RMS roughness (squares) from the AFM measurements and friction coefficients (triangles) from the FFM analysis contrasted to the adsorbed amount based on SPR analyses.

Cellulose in general is not very prone to non-specific protein adsorption. This originates from the highly hydrated, hydrophilic cellulosic material, having hydrogel characteristics. Upon protein deposition, the water that is close to the surface of the cellulose and on the surface of the protein needs to be replaced—a process which is, if there are not any specific contributions—entropically unfavorable. Several approaches have shown that either anionic or cationic coatings on cellulose thin films may alter the adsorption behavior of proteins (Orelma et al., 2011; Mohan et al., 2013, 2014) depending on the employed pH during adsorption and the isoelectric point of the protein. On hydrophobic surfaces, proteins adhere spontaneously in a non-specific fashion and often they (partially) lose their ternary or quaternary structure and denaturate during deposition (Norde and Lyklema, 1991; Sagvolden et al., 1998). One would expect that the incorporation of a hydrophobic component such as HPCE into the cellulose film would trigger non-specific adsorption different from affinity to the cellulose regions. We chose BSA (pH 7.4) as a demonstrator probe for non-specific protein interactions of the blend films and studied the adsorption process using a combination of SPR spectroscopy and QCM-D. The combination of these techniques gives complementing insight into the amount of adsorbed protein. While QCM-D is a gravimetric technique capable of sensing any type of mass (i.e., water, and BSA, Γ^QCM^) on the surface, SPR spectroscopy allows for determination of dry mass (Γ^SPR^) by employing the de Feijter equation (1). The difference between Γ^QCM^ and Γ^SPR^ is the amount of water in the film. This amount of water decreased with lowering the cellulose content in the films and reaches its minimum for the 1:3 cellulose:HPCE film (Figure 6A). The HPCE film should swell the least, and hence, the major fraction of water can be assumed to be associated with the BSA on the surface. The amount of water in the BSA layer is 65%. The HPCE film experiences the highest BSA adsorbed amount (based on SPR) of 1 mg m^−2^. The other extreme is the neat cellulose film that features low BSA adsorption (Γ^SPR^ 0.1 mg m^−2^) which is in good agreement with other reports in these conditions (Niegelhell et al., 2017; Weißl et al., 2018). The highly swollen hydrogel character is revealed in the QCM-D data for the pure cellulose film, showing 1.7 mg m^−2^ water in the layer corresponding to 96% of hydration.

The increase in volume fraction of the HPCE led to a linear increase in the amount of adsorbed BSA on the surfaces. Contrasting the SPR adsorbed mass to the effective surface fraction revealed that the 1:1 blend film (volumetric) was an outlier in the adsorption trend and a lower amount of protein was deposited than what would have been expected (Figure 6B). Comparing this data to the other few reports on protein adsorption on cellulose based blend films revealed that also for the other systems protein adsorption minima were determined close to the 1:1 volume ratio (Niegelhell et al., 2016, 2017; Strasser et al., 2016). The cellulose:HPCE 3:1 film contains 57% of cellulose based on surface fraction, but still, it shows a higher BSA adsorption (0.39 mg m^−2^) than the cellulose:HPCE 1:1 film (0.30 mg m^−2^) with lower cellulose content (37% surface fraction).

The influence of surface free energy, roughness, and friction coefficient on the BSA adsorption is plotted against $\Gamma_{,}^{\text{QCM}}$ $\Gamma_{,}^{\text{SPR}}$ and bound water (Figures 6C,D). The SFE has a decisive influence on the BSA adsorption (Figure 6C). For the neat cellulose and HPCE films, surface roughness as well as friction coefficient were similar, but the SFE significantly differed. For the blend films however, the correlation between SFE and adsorbed mass was straightforward at first glance: lower surface energy translated to more hydrophobic surface resulting in higher non-specific protein deposition (Figure 6C). However, the SFE development did not correlate linearly with the effective surface fraction (Figure S3). There is a plateau in the SFE development with surface fraction of 0.37 (1:1) after which the cellulose dominated the SFE. One would expect much lower SFEs due to the actual surface fraction of just 37% of cellulose which would lead to higher non-specific protein adsorption. It is therefore rather the wetting properties of the blend films that are tuned by the morphology and configuration and these further affect the adsorption. The roughness did not have a direct correlation to the adsorbed mass through the series, and neither did the friction coefficient (Figure 6D). Since the adsorbed mass and the SFE exhibited a linear correlation, it is clear that the roughness and friction coefficient correlation with SFE is similar to the adsorbed mass (Figure S5 vs. Figure 6D).

## Conclusions

Friction of cellulose films could be altered by blend composition including a low surface energy derivative. A surface fraction of 37% of cellulose (1:1 volume fraction) exhibited outlier adsorption inhibiting the protein adsorption. Surface energy rather than friction was a decisive factor for protein adsorption on the films. However, the SFE did not follow linearly the component surface area fraction. Wetting and adhesion typically correlate with surface friction. However, this correlation was not verified here to provide a correlation of friction to protein adsorption on the surface. Surface configuration—periodicity of the structure and feature size—might be an underlying factor.

## Author Contributions

CC was responsible of FFM and some of the AFM measurements, FFM data analysis, area calculations, and contributed in writing of the manuscript. GT was responsible of the SPR and QCM-D measurements, area calculations, and writing. MN and MB contributed with HPCE synthesis and characterization and MH with AFM measurements. CP assisted with QCM-D measurements and corresponding data analysis. SS and CT contributed in data analysis and writing. TN contributed with experimental analyses and writing the manuscript.

### Conflict of Interest Statement

The authors declare that the research was conducted in the absence of any commercial or financial relationships that could be construed as a potential conflict of interest.
