# Supplementary material for: Design of Friction, Morphology, Wetting, and Protein Affinity by Cellulose Blend Thin Film Composition
Source: Front Chem. 2019 May 3;7:239. doi: 10.3389/fchem.2019.00239 (PMC6509480; doi:10.3389/fchem.2019.00239)
Supplement: Supplementary file 1 [file Data_Sheet_1.PDF]

Supporting information file

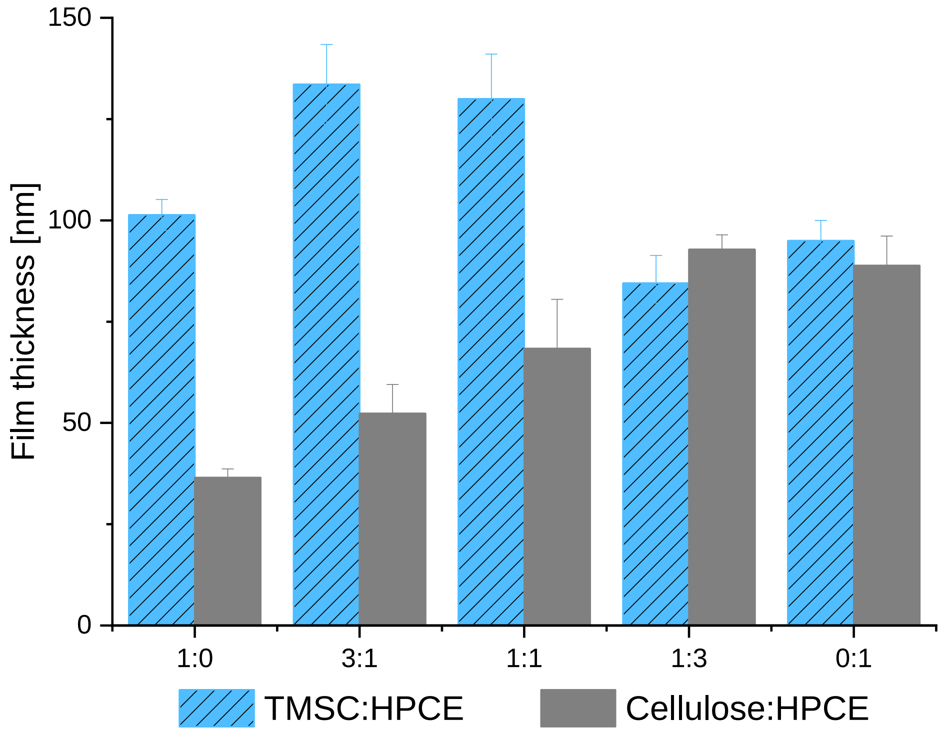

Figure S1. Blend film thickness determined by profilometer before (blue textured) and after regeneration (grey solid).

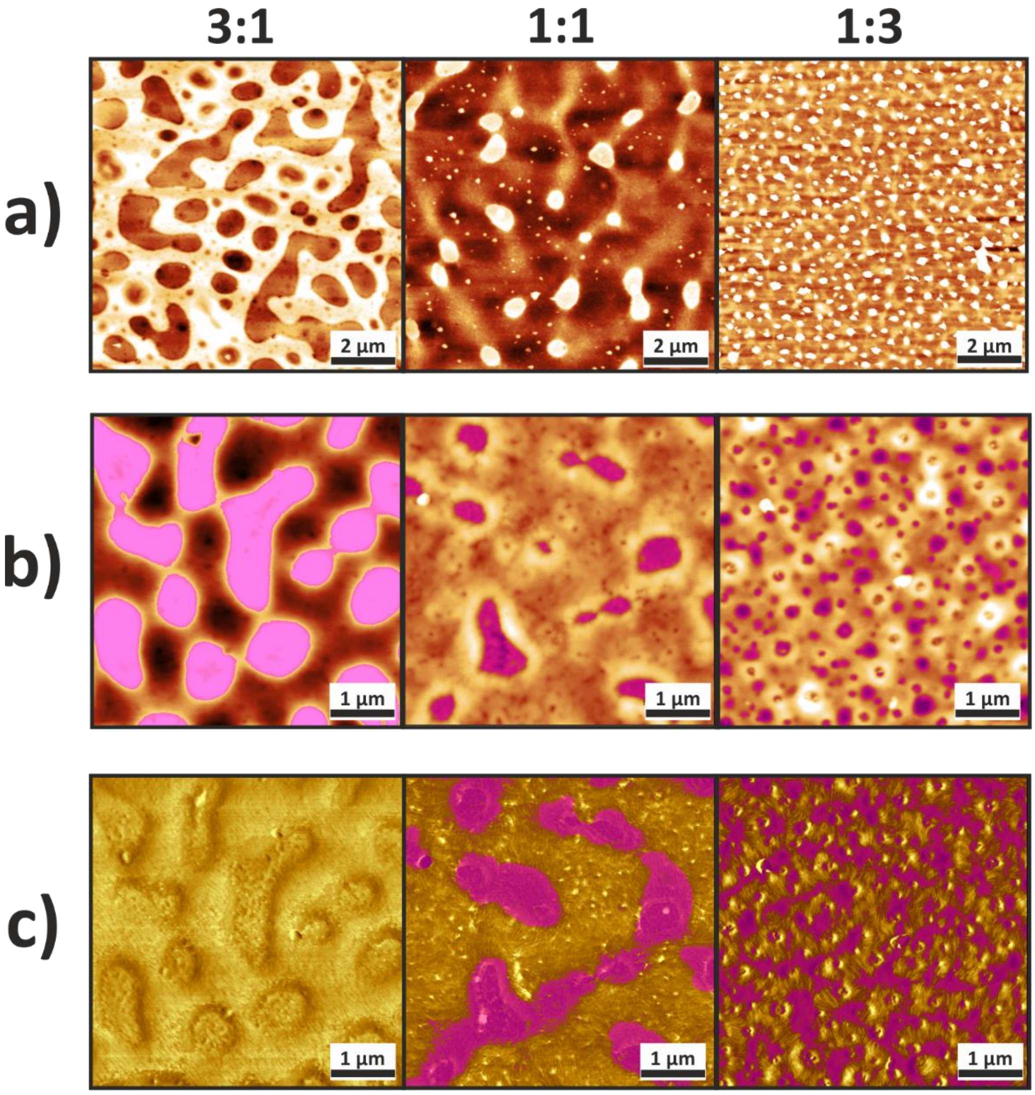

Figure S2. (a) AFM topography images of the blend films after rinsing the films with chloroform. (b) AFM topography, (c) and phase images with masking applied to identify surface fractions of the phases.

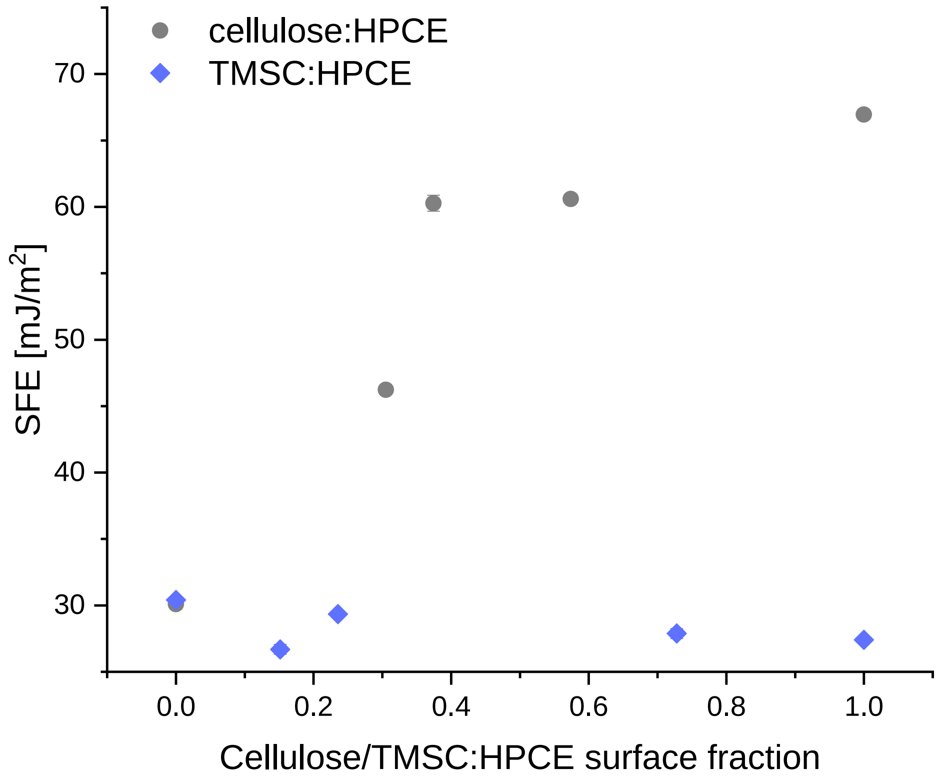

Figure S3. Surface free energy of the cellulose:HPCE (circles) and TMSC:HPCE (diamonds) blend films as a function of surface fraction.

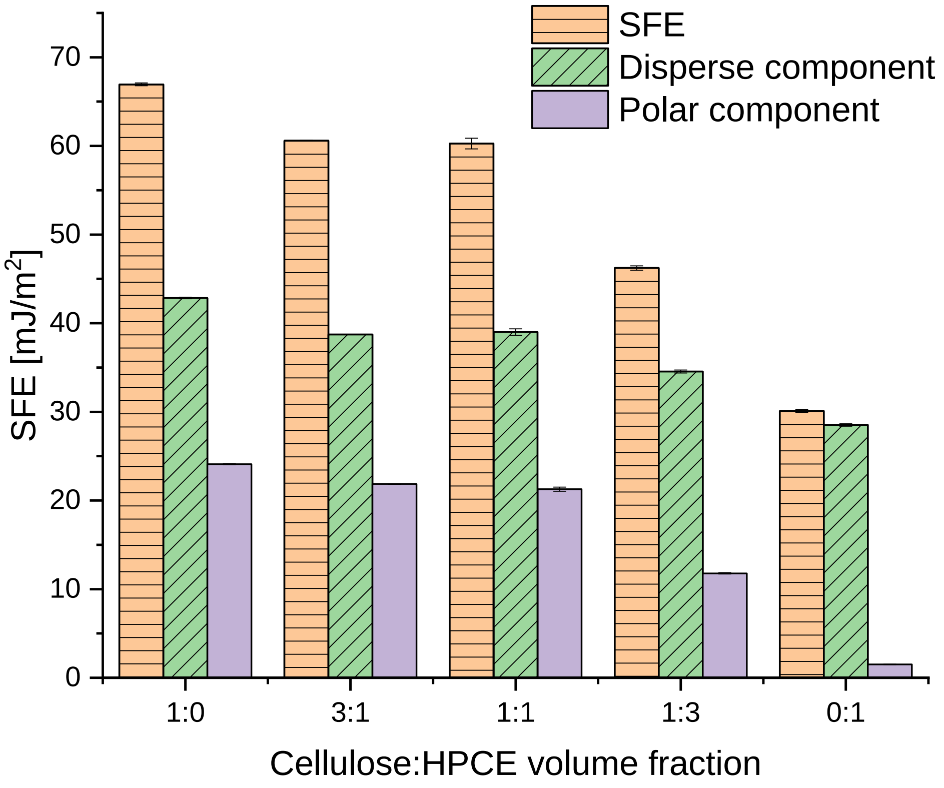

Figure S4. Disperse (diagonal lines) and polar (no texture) components of the SFE (horizontal lines) of cellulose/HPCE blend films with various volume fractions.

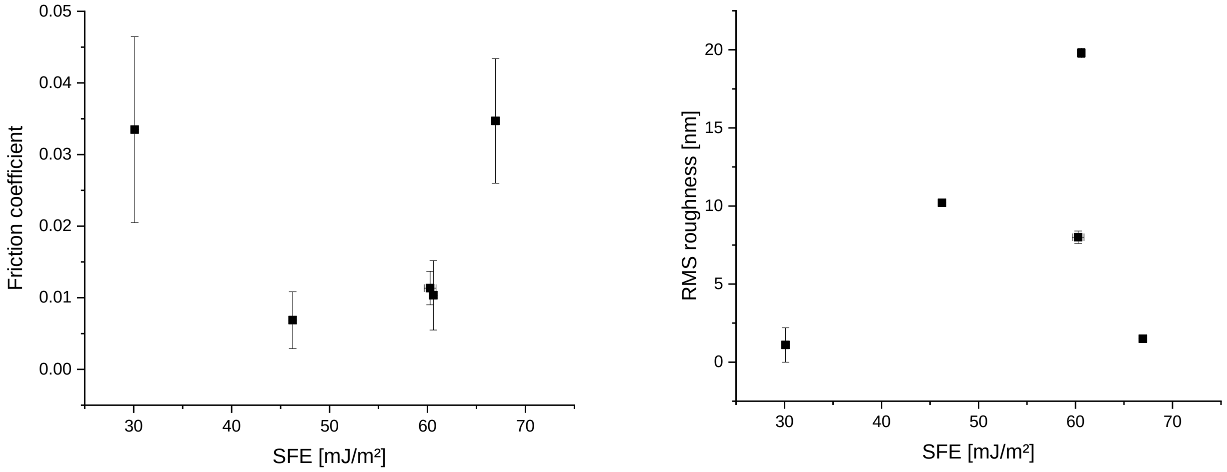

Figure S5. Friction coefficient (left) and RMS roughness (right) of the blend films contrasted to the surface free energy.
